# How I do it: a simulator of the ear for developing otomicroscopy skills during the coronavirus disease 2019 pandemic

**DOI:** 10.1017/S0022215120002261

**Published:** 2020-10-21

**Authors:** C Shenton, W Aucott

**Affiliations:** Otolaryngology Department, Blackpool Victoria Hospital, UK

**Keywords:** Ear, Otologic Surgical Procedures, Otolaryngology, Coronavirus disease 2019, Motor Skills, Education

## Abstract

**Objective:**

To develop a simulator of the external auditory canal and tympanic membrane that enables surgical trainees to practise their otomicroscopy skills, which is particularly valuable at a time where there is limited patient contact because of the coronavirus disease 2019 lockdown.

**Methods:**

A simulator of the external auditory canal and tympanic membrane was made using a cardboard bowl, a 2 ml syringe and a latex glove. The simulator was used to practise otomicroscopy skills, including microsuction, foreign body removal, myringotomy and grommet insertion. Five doctors in the ENT department participated, ranging from core surgical training year two doctor to specialty doctor.

**Results:**

The simulator provides an effective tool on which surgical trainees can practise, develop and maintain a variety of otomicroscopy skills.

**Conclusion:**

This inexpensive, easy and quick-to-make simulator enables trainees to practise their otomicroscopy skills on an approximately accurate model during a time when there is minimal clinical opportunity to develop these skills, particularly because of the coronavirus disease 2019 pandemic.

## Introduction

The coronavirus disease 2019 (Covid-19) pandemic has resulted in the postponement of non-urgent operations and procedures in the UK because of the risk to patients and staff. Regarding ENT, such procedures include myringotomy, grommet insertion, aural microsuction, otomicroscopy and removal of aural foreign bodies (excluding button batteries).^1^ These skills are a desired competency in the UK for trainees wanting to pursue a career in ENT.^2,3^ We therefore developed a simple simulator of the ear, to enable surgical trainees to practise or maintain these skills whilst the clinical opportunity to do so is currently limited. This simulator is grossly anatomically accurate, and quick and easy to make.

## Materials and methods

Five doctors in the ENT department participated, ranging from core surgical training year two doctor to specialty doctor.

**Fig. 1.**
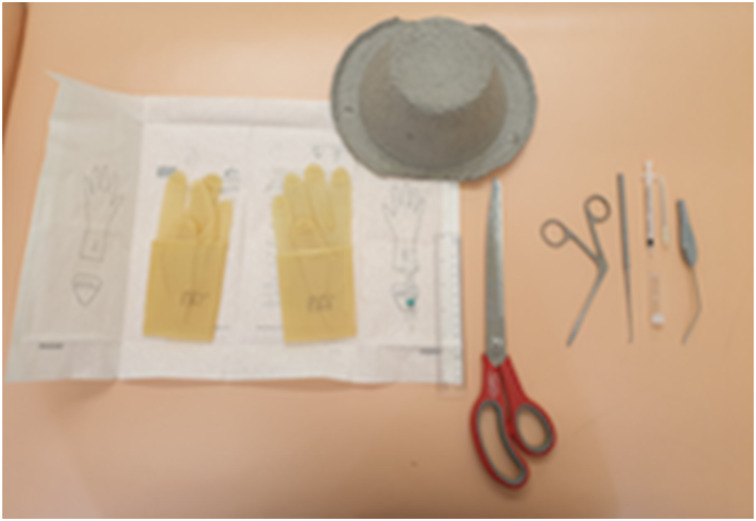
Equipment required: latex glove, cardboard bowl and 2 ml syringe.

**Fig. 2.**
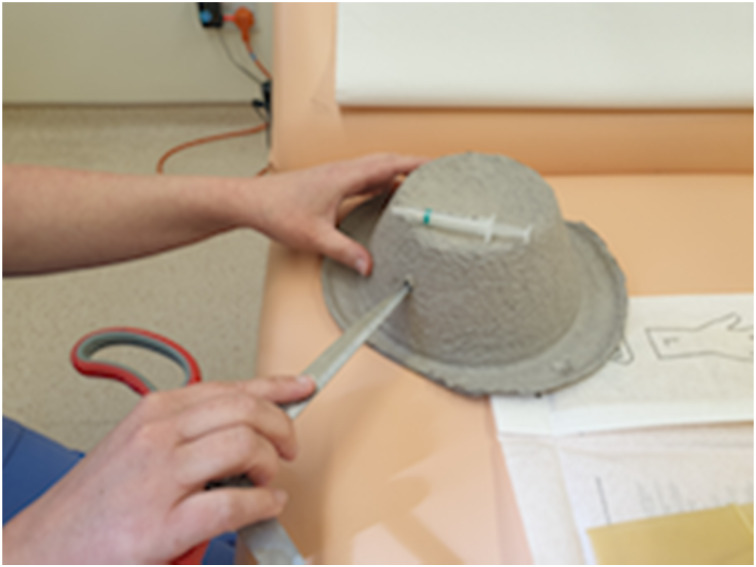
Make a hole in the cardboard bowl (the head).

**Fig. 3.**
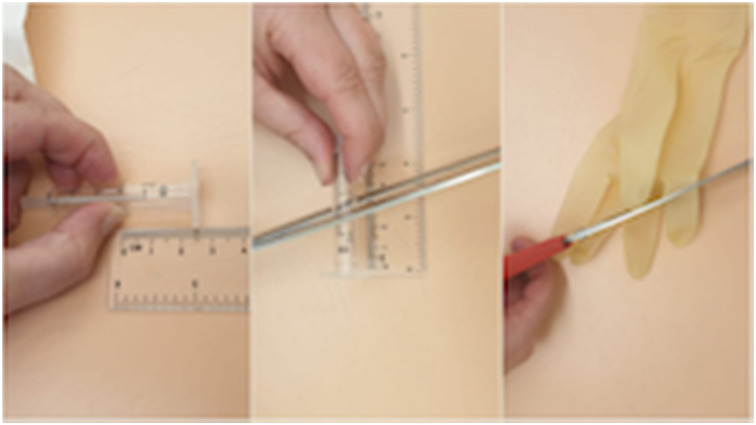
For the external auditory canal and tympanic membrane, cut the syringe at an angle, to a length of 2.5 cm, and cut a finger off the glove.

Our simulator consists of a cardboard bowl, a 2 ml syringe and a latex glove (Figure 1). A hole the diameter of the 2 ml syringe is made in the bowl (Figure 2). The syringe is cut at a slight angle, to a length of 2.5 cm from the open end. A finger is then cut off the glove (Figure 3).

**Fig. 4.**
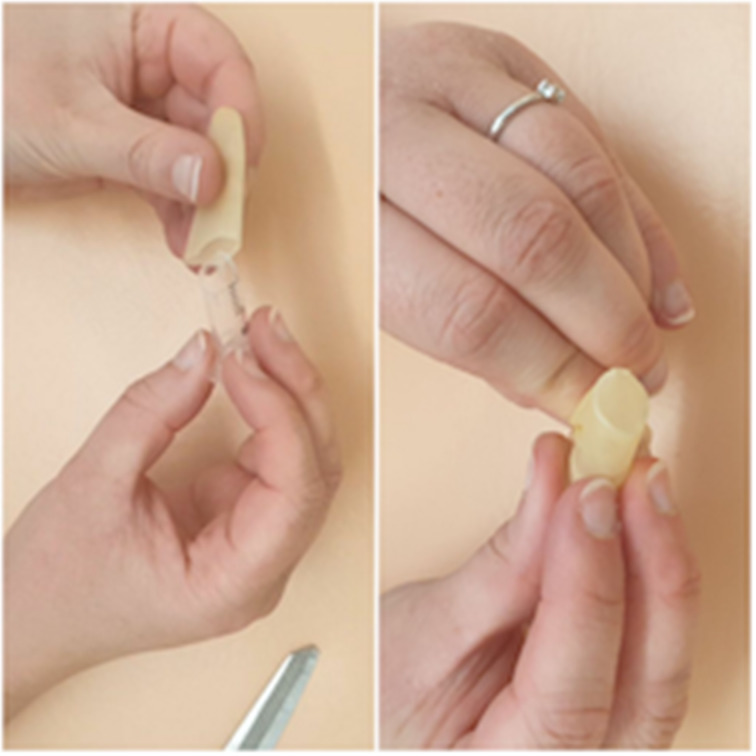
Insert syringe into glove until the glove is taut.

**Fig. 5.**
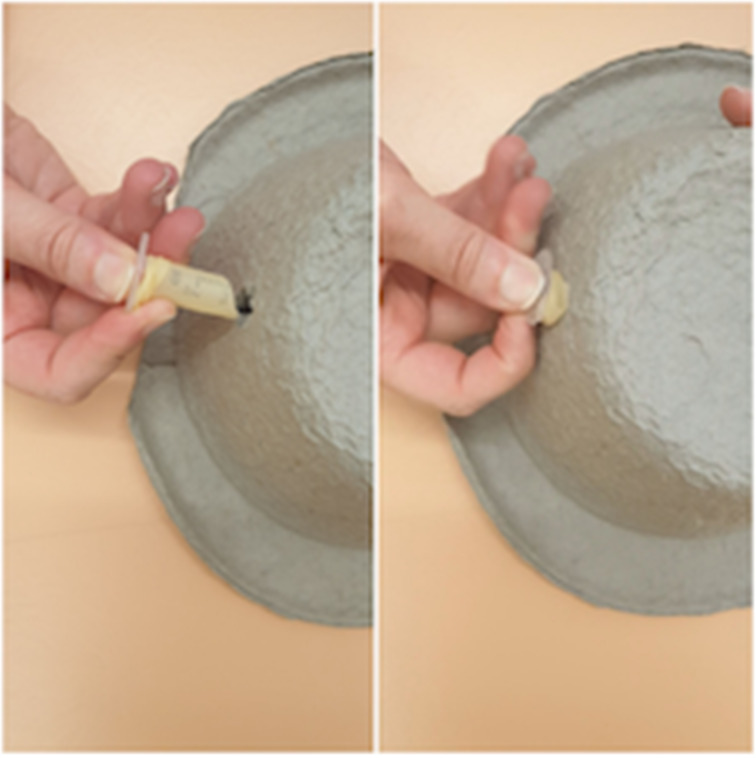
Insert syringe into hole in bowl.

**Fig. 6.**
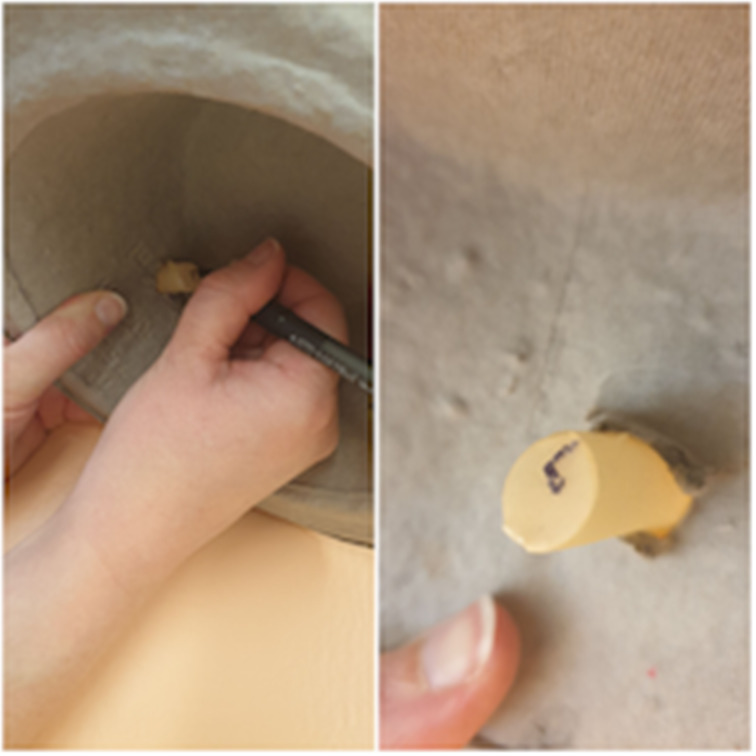
Draw a handle of malleus on the glove.

The cut end of the syringe is inserted into the cut finger of the glove until the glove creates a taut surface at the angled end of the syringe; this represents the tympanic membrane (Figure 4). The syringe is inserted into the hole in the bowl (Figure 5). A handle of malleus is drawn on the glove with a ballpoint pen (use the cut angle of the syringe to determine which part of the ‘tympanic membrane’ is anterior and which is posterior) (Figure 6).

**Fig. 7.**
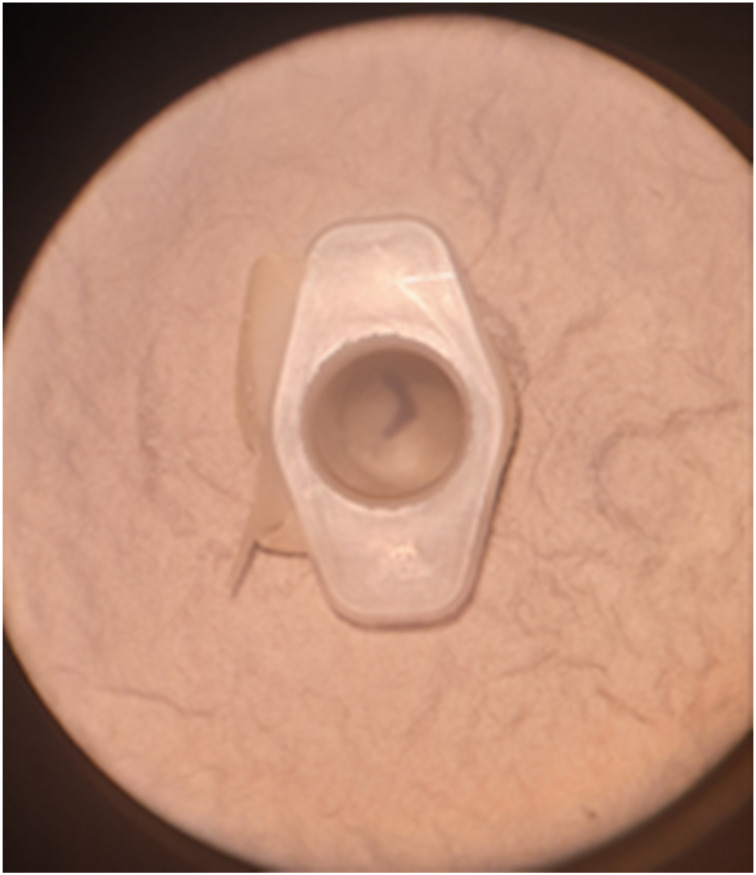
External auditory canal and tympanic membrane viewed under microscope.

**Fig. 8.**
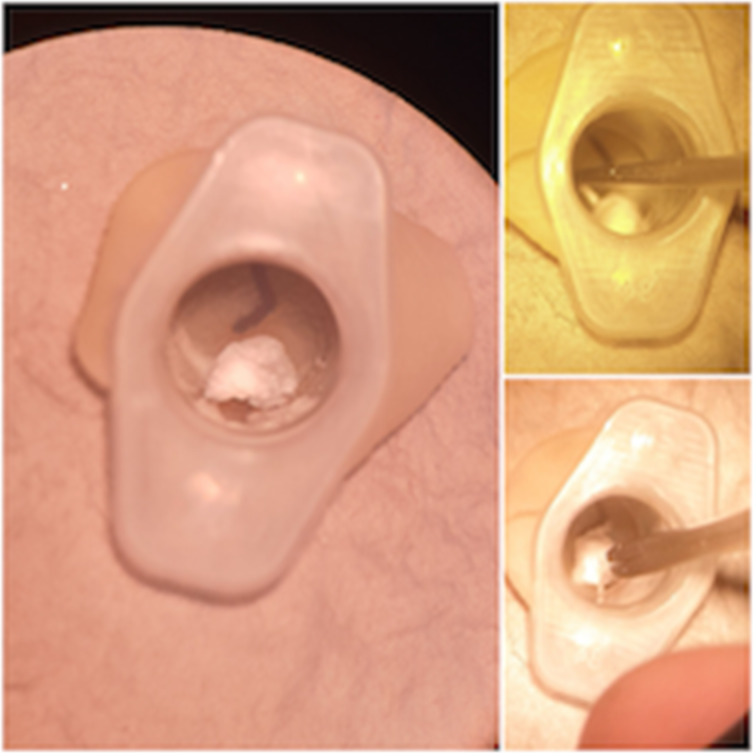
Foreign body, removed with hook and forceps.

**Fig. 9.**
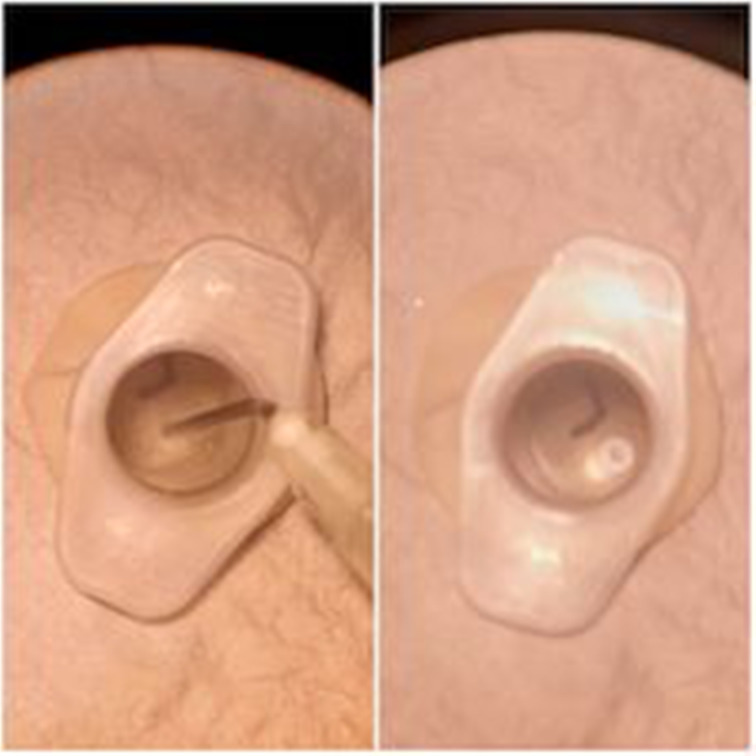
Myringotomy and grommet insertion.

The simulator can be used with an otoscope or a microscope (Figure 7). A wet ball of tissue was used to simulate a foreign body, with blue tac as a substitute for ear wax. Crocodile forceps, a wax hook and microsuction were used to practise foreign body removal (Figure 8). We used a white needle on the end of a 1 ml syringe as a myringotomy blade, and a demonstration grommet to practise myringotomy and grommet insertion (Figure 9).

## Results

### Model benefits

The simulator is made from materials that are readily and cheaply available. It requires no special skills to assemble. Furthermore, it is easily transported and stored. Participant feedback suggests that making it is easy and takes 5–10 minutes. It is also reproducible, and one glove can be used to make several tympanic membranes.

As shown in Figure 8, the simulator can be used to practise otomicroscopy skills, including microsuction and foreign body removal, with a variety of instruments.

Using a latex glove as the tympanic membrane provides a surface with similar tension to the tympanic membrane. This enables clinicians to practise myringotomy and grommet insertion on a simulator with similar tactile feedback to an actual tympanic membrane (Figure 9).

The participants found the simulator to be as reported above, and thought that it represented the tympanic membrane and external auditory canal well.

### Model limitations

The maker of the simulator should ensure that the handle of the malleus is correctly drawn and the syringe is inserted in the correct orientation to accurately represent the anatomy, with the acute angle anteriorly.

Regarding myringotomy practice, the needle has an angled blade, which makes controlling the incision harder compared to using a myringotomy blade. This could be overcome by investing in a myringotomy blade, but some surgeons do often use a needle or Venflon cannula.

Usually the simulator would be used with the clinic or ward microscope, where access to a range of instruments allows full use of this model.

## Discussion

Three similar do-it-yourself models exist in the literature. The first uses a plastic egg box, modelling clay, a 3 cc syringe and micropore tape (latex gloves were suggested as an alternative for the tympanic membrane, along with paraffin or cellophane).^4^ Our simulator uses different and fewer materials that are all readily available in a hospital setting. The second model uses a paper kidney basin, plastic blood vials and tape.^5^ The third model uses two disposable auricular temperature probe covers.^6^ Neither of the latter two models incorporates an external auditory canal. Our use of a 2 ml syringe creates a grossly anatomically accurate external auditory canal, enabling practice of a variety of otomicroscopy skills on a more anatomically similar model.

More complex models also exist. These use a variety of materials, including: a wooden base, tape and 5 ml syringes;^7^ silicone sealant, aluminium tubing and plastic sheets;^8^ wood, polyester filling, a solution bottle's cap and polyethylene film;^9^ a Styrofoam head, a T-pin malleus, a syringe and glad wrap;^10^ and polyvinyl chloride, clay, acrylonitrile butadiene styrene and clear plastic.^11^ In comparison with our simulator, these models take longer to assemble, and require more specialist skills (such as soldering) and more expensive materials that are not readily available in clinical practice.^7,8,10,11^

Virtual reality simulators have also been developed;^12–14^ these are beyond the reach of a trainee.

## Conclusion

We have made a simple, inexpensive and easily reproducible model of the external auditory canal and tympanic membrane that provides trainees with an opportunity to safely practise their otomicroscopy skills. This is very relevant during the current Covid-19 pandemic, where trainees have limited clinical opportunities to develop or maintain these essential skills. The simulator allows skill acquisition and development, at any time, under less pressure, and without any risk to patients.

Although similar models exist, we have created a quick and easy-to-make, grossly anatomically accurate ear simulator, using only three materials, which are readily available in clinical practice. We feel this model is easier and more likely to be attempted than other designs we have found.
